# Association between Systemic Immune-Inflammation Index (SII) and New-Onset In-Hospital Heart Failure in Patients with STEMI after Primary PCI

**DOI:** 10.31083/j.rcm2510382

**Published:** 2024-10-24

**Authors:** Huibo Wang, Ying Yang, Ping Zeng, Rihong Huang, Xinyong Cai, Liang Shao, Fuyuan Liu, Yuhua Lei, Dongsheng Li, Zhixing Fan, Jun Yang, Jing Zhang, Jian Yang

**Affiliations:** ^1^Department of Cardiology, The First College of Clinical Medical Science, China Three Gorges University and Yichang Central People’s Hospital, 443000 Yichang, Hubei, China; ^2^Department of Cardiology, Institute of Cardiovascular Diseases, China Three Gorges University, 443000 Yichang, Hubei, China; ^3^Department of Cardiology, Hubei Key Laboratory of Ischemic Cardiovascular Disease, 443000 Yichang, Hubei, China; ^4^Department of Cardiology, Hubei Provincial Clinical Research Center for Ischemic Cardiovascular Disease, 443000 Yichang, Hubei, China; ^5^Department of Cardiology, Renmin Hospital of Wuhan University, 430060 Wuhan, Hubei, China; ^6^Department of Cardiology, First Affiliated Hospital of Dalian Medical University, 116021 Dalian, Liaoning, China; ^7^Department of Cardiology, Jiangxi Provincial People’s Hospital Affiliated to Nanchang University, 330038 Nanchang, Jiangxi, China; ^8^Department of Cardiology, The No1. People’s Hospital of Xiangyang, 441099 Xiangyang, Hubei, China; ^9^Department of Cardiology, The Central Hospital of Enshi Tujia and Miao Autonomous Prefecture, 445099 Enshi, Hubei, China; ^10^Department of Cardiology, Wuhan Third Hospital & Tongren Hospital of Wuhan University, 430060 Wuhan, Hubei, China

**Keywords:** systemic immune-inflammation index, heart failure, ST-segment elevation myocardial infarction, primary PCI

## Abstract

**Background:**

The systemic immune-inflammation index (SII) is a proven, reliable inflammatory marker of the atherosclerotic process. Additionally, inflammation is one of the most important mechanisms of heart failure (HF) after myocardial infarction (MI). However, it is not clear whether SII is related to the risk of in-hospital HF in patients with MI. Thus, we aimed to explore the relationship between SII and the risk of new-onset in-hospital HF in ST-segment elevation myocardial infarction (STEMI) patients treated with primary percutaneous coronary intervention (pPCI).

**Methods:**

A total of 5586 patients with STEMI underwent pPCI at seven clinical sites in China from January 2015 to August 2021. The patients were divided into two groups based on the SII values. The association between SII and new-onset in-hospital HF in STEMI patients was assessed using logistic regression analysis.

**Results:**

Ultimately, 3808 STEMI patients with Killip class I who were treated with pPCI were included. All included patients were divided into two groups based on the calculated SII (Q1 SII: <1707.31 (×10^9^/L), Q2 SII: ≥1707.31 (×10^9^/L)). After unadjusted and multivariate adjustment for age, gender, vital signs, smoking, hypertension, diabetes mellitus, *etc*., the odds ratio (OR) of the in-hospital HF risk in Q2 was 1.378–1.427 times the Q1 in the calibration Models 1 to 5. Subgroup analysis showed that the OR of Q2 was 1.505-fold higher of Q1 in males and 1.525-fold in older people (≥60 years). Sensitivity analysis showed that after excluding patients who had previously experienced HF, MI, or underwent PCI, elevated SII was still associated with a significant increase in the risk of in-hospital HF.

**Conclusions:**

Elevated SII is associated with an increased risk of in-hospital HF in STEMI patients treated with pPCI, particularly in male and older patients.

**Clinical Trial Registration:**

The Chinese STEMI pPCI Registry was registered with ClinicalTrials.gov (NCT04996901, https://www.clinicaltrials.gov/study/NCT04996901?cond=NCT04996901&rank=1).

## 1. Introduction

Most coronary artery disease (CAD) refers to coronary atherosclerotic lesions that result in severe vascular narrowing or obstruction, leading to inadequate myocardial blood supply or necrosis [1]. Acute myocardial infarction (AMI), which has traditionally been divided into ST elevation (STEMI) or non-ST elevation myocardial infarction (NSTEMI), is the most acute manifestation of CAD and is associated with great morbidity and mortality [1, 2]. Predominantly, STEMI is caused by complete thromboembolism resulting from an atherosclerotic plaque in the epicardial coronary artery [2]. Percutaneous coronary intervention (PCI) is the preferred and most reliable treatment method for restoring myocardial perfusion using cardiac catheterization technology [3]. Despite the remarkable advances in treating CAD and AMI over the past two decades, AMI remains the most common cause of heart failure (HF) [4, 5]. Since impaired coronary reflow is accompanied by HF and mortality in AMI, knowing the potential mechanism and predicting this situation has become imperative.

Study has evidenced that inflammation is doubtless the main reason for the occurrence and maintenance of atherosclerosis, including CAD [6]. Meanwhile, the systemic immune-inflammation index (SII) is an integrated and novel inflammatory marker associated with the development of atherosclerosis and the resulting stability of CAD [7]. It has been suggested that since this formula includes three inflammatory cell types (platelet, neutrophil, and lymphocyte), it can be calculated via platelet count × neutrophil count/lymphocyte count [8, 9]. Inflammation has been proven to be associated with STEMI and subsequent new-onset HF; however, the relationship between the new composite inflammatory level indicators SII and STEMI has not been clearly defined. Thus, determining this relationship is important since it would allow the prediction of new-onset in-hospital HF, thereby reducing hospital stays and mortality. Therefore, we hypothesize that SII is related to new-onset in-hospital HF in STEMI patients undergoing primary PCI (pPCI). Hence, the present study aimed to investigate the predictive value of inflammatory marker SII for new-onset in-hospital HF in patients with STEMI undergoing pPCI.

## 2. Methods

### 2.1 Data Availability

The authors declare that all supporting data are available from the corresponding author upon reasonable request.

### 2.2 Study Population and Design

This retrospective observational study was registered in Clinical Trials (NCT04996901) and conducted at 7 clinical sites in China. All eligible patients were diagnosed with STEMI within 24 hours of the onset of symptoms and underwent pPCI between January 2015 and August 2021. STEMI was defined as prolonged typical chest pain for more than 20 minutes with electrocardiogram characteristics of sustained ST elevation in no less than 2 continuous leads or a new left branch bundle block pattern in accordance with the criteria specified in the guidelines [2]. Patients admitted for the first time or transferred from another hospital were included in this study.

**Fig. 1.**
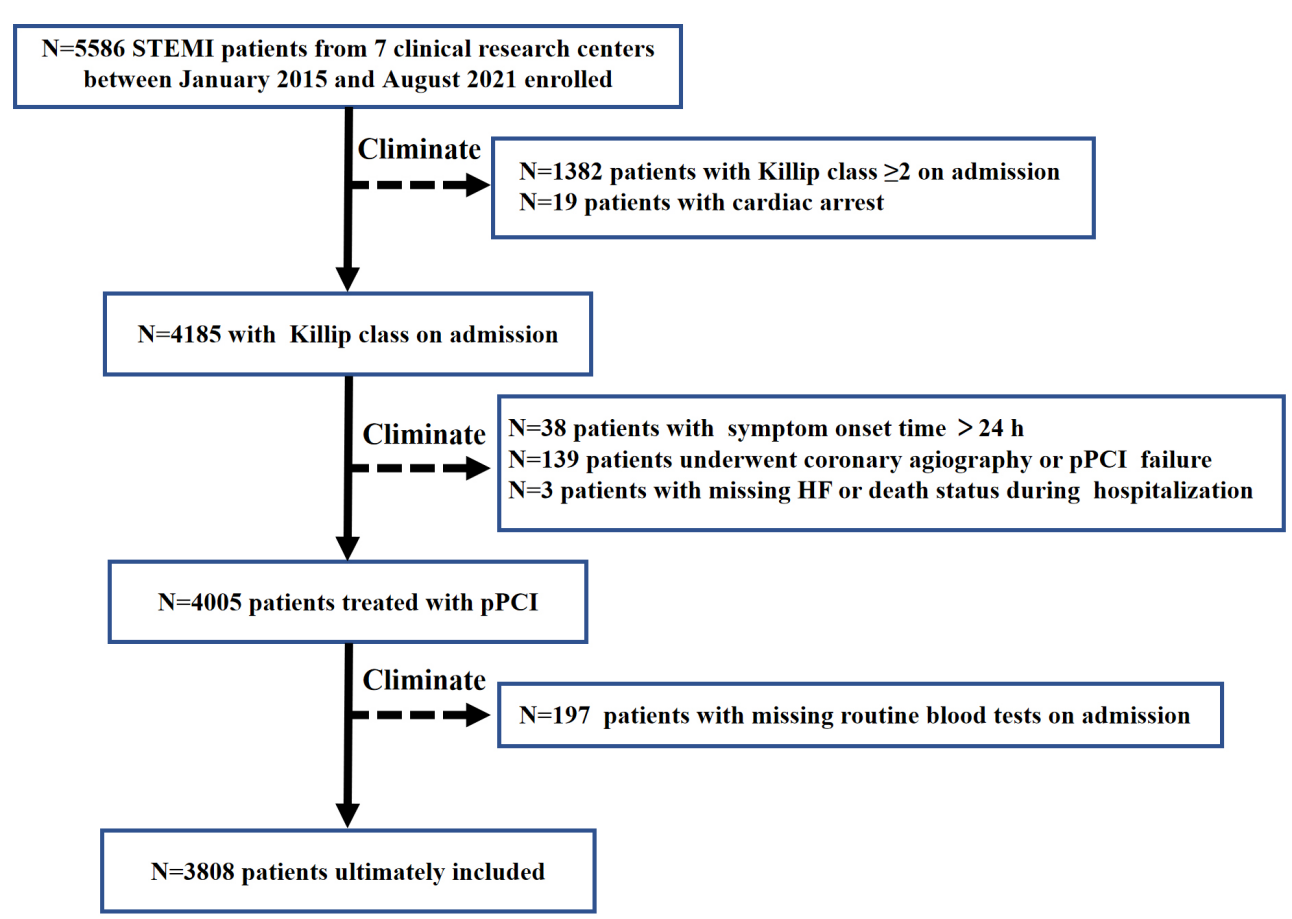
**Flowchart of the patient screening process in this study**. STEMI, ST-segment elevation myocardial infarction; pPCI, primary percutaneous coronary intervention; HF, heart failure.

A total of 5586 STEMI patients treated with pPCI were included in this study. Patients with available routine blood tests who reported on the first day of hospitalization were enrolled. The exclusion criteria included: Killip class ≥2 on admission or those undergoing cardiac arrest; symptom onset time beyond 24 h; without or failure of pPCI treatment; missing cardiac function or routine blood tests on admission and other information. Finally, 3808 participants were eligible for inclusion to assess the association between SII and the risk of new-onset in-hospital HF (Fig. 1). This study was approved by the Ethics Committee of Renmin Hospital of Wuhan University (NO: WDRY2021-K054) and the Ethics Committee of Yichang Central People’s Hospital (NO: 2021-043-01), and complied with the Helsinki Declaration.

### 2.3 Clinical Data Collection

Blood samples were collected by venipuncture before the procedure as part of routine patient care, and complete blood count parameters, stored in heparinized tubes, were measured by standard technique before pPCI. SII was calculated by platelet count × neutrophil count/lymphocyte count, both obtained from the same blood sample drawn on admission [8, 9]. All data were obtained from the digital information systems of 7 participating clinical hospitals (Renmin Hospital of Wuhan University; The First College of Clinical Medical Science, China Three Gorges University; The Central Hospital of Enshi Tujia And Miao Autonomous Prefecture; Xiangyang No.1 People’s Hospital; Wuhan Third Hospital; The First Affiliated Hospital of Dalian Medical University; Jiangxi Provincial People’s Hopital). Demographic information, previously associated diseases (diabetes, various types of cardiovascular disease), life factors (e.g., smoking), laboratory test results (e.g., routine blood tests), the Killip class, data on vital signs (e.g., diastolic blood pressures (DBP), systolic blood pressures (SBP), and heart rate (HR)), medication situation and PCI data were collected. The blood flow grade of thrombolysis in myocardial infarction (TIMI) was obtained from angiographic data. TIMI (ranging from 0 to 3) is a semi-quantitative blood flow index based on vessels with significant stenosis [10].

### 2.4 Emergency Coronary Angiography and PCI

All enrolled patients were treated with pPCI as soon as possible and administered aspirin (300 mg), clopidogrel (300–600 mg)/ticagreloras (180 mg) adjuvant antiplatelet therapy, and anticoagulant therapy with unfractionated heparin (3000 IU) before the coronary angiography (CAG). Unfractionated heparin was supplemented to 100 IU/kg before pPCI according to body weight, and tirofiban was routinely used for enhanced antiplatelet therapy after surgery. The CAG results were determined by at least two experienced cardiovascular interventional physicians. The interventional doctors recommended the optimal treatment strategies to patients based on the angiography results, according to the current practice guidelines [11].

### 2.5 Definitions

New-onset HF was defined as a Killip ≥2 class after hospitalization, the first sign of bilateral rales or pulmonary edema after the initial clinical assessment [7]. Assessments of terminal status were conducted by the designated physicians based on medical records: (1) high flow rate oxygen inhalation is required, including the use of invasive or non-invasive respirators to maintain oxygen saturation; (2) requiring inotrope or mechanical support such as intra-aortic balloon pump (IABP) or extracorporeal membrane oxygenation (ECMO); (3) requiring various types of diuretics [12]. SII was calculated by (neutrophil counts) × (platelet counts)/(lymphocyte counts) [8, 9]. The included patients were divided into two groups based on the calculated SII values: Q1 SII: <1707.31 (×10^9^/L) and Q2 SII: ≥1707.31 (×10^9^/L).

### 2.6 Statistical Analysis

All statistical analyses were performed using R software (4.2.1, R Foundation for Statistical Computing, Vienna, Austria). Continuous variables in accord with a normal distribution were expressed as the mean ± standard deviation; otherwise, they were presented as the median (P25, P75); categorical variables are reported as numbers (percentages). The between-group differences were evaluated by an independent-sample *t*-test for normal distribution data; meanwhile, the Mann-Whitney U test was used for non-normal data. The association between different groups of SII levels and the risk of new-onset in-hospital HF was assessed in an adjusted logistic regression model, in which Q1 (SII: <1707.31 × 10^9^/L) served as a reference.

Many factors may affect the correlation between SII and the risk of new-onset in-hospital HF, so the multivariate test was built using five models to eliminate their influence: Model 1 with no variables adjusted; Model 2 was adjusted for age, gender, SBP, DBP, and HR; Model 3 was further adjusted for historical occurrences (smoking, diabetes mellitus, hypertension, hyperlipidemia, atrial fibrillation, and previous stroke) based on Model 2; Model 4 was further adjusted for history of present status (symptom onset to balloon time, preoperative malignant arrhythmia, syncope) based on Model 3; Model 5 was further adjusted for pPCI treatment status (TIMI flow grade 0 before pPCI, TIMI flow grade 3 after pPCI, non-infarct related artery lesions PCI management, multivessel disease, infarct-related artery lesions PCI management) based on Model 4; analysis of *p* for trend was conducted in the form of the median. Subgroup analyses of gender and age were performed based on Model 5. Sensitivity analysis was conducted after excluding the patients who previously had HF and MI. Receiver operating characteristic (ROC) curve analysis and area under the curve (AUC) calculations were used to test model differentiation and calibration degree. Statistical significance was determined by *p* < 0.05.

## 3. Results

### 3.1 Flowchart of Patient Screening Process

During the study period, between January 2015 and August 2021, a total of 5586 patients with STEMI were enrolled in our registry. Among them, we excluded 1382 patients with Killip class ≥2 on admission and another 19 patients with cardiac arrest. Additionally, 38 patients with symptoms that appeared more than 24 hours after admission, 139 patients with failed or without pPCI treatment, and 3 patients with missing HF or death status during hospitalization were excluded. Finally, 197 patients without routine blood tests on admission were also excluded. Overall, a total of 3808 patients were included (Fig. 1).

### 3.2 Baseline Characteristics

The baseline characteristics of the studied groups are presented in Table 1. Detailed information was collected on all included STEMI participants, including sociodemographic characteristics (age, gender), vital signs (SBP, DBP, HR), lifestyle factor (smoking), and past medical history (hypertension, diabetes mellitus (DM), hyperlipidemia, atrial fibrillation (AF), HF, MI, PCI, stroke). No significant differences were found in the demographic characteristics (age, gender), DBP, lifestyle factor (smoking), and most past medical history (hypertension, hyperlipidemia, previous AF, previous HF, previous MI, previous PCI, previous stroke). The average SBP (124.00 (110.00, 140.00) *vs*. 122.00 (110.00, 138.00), *p* = 0.008) and the proportion of patients with DM (458 (19.00%) *vs*. 213 (15.3%), *p* = 0.004) in Q2 group were higher than those in Q1 group, but the average HR (75.00 (66.00, 85.00) *vs*. 78.00 (70.00, 87.00), *p* < 0.001) was lower than that in Q2 group.

**Table 1.** **Baseline characteristics according to SII level strata**.

	Terms	Overall	Q1 (<1707.31)	Q2 (≥1707.31)	*p*-value
		3808	2412	1396	
Demography	Age (y)	60.00 (51.47, 68.00)	60.00 (52.00, 69.00)	60.00 (51.00, 68.00)	0.089
	Female (%)	681 (17.90)	425 (17.60)	256 (18.30)	0.577
Vital signs	SBP (mmHg)	123.00 (110.00, 139.00)	124.00 (110.00, 140.00)	122.00 (110.00, 138.00)	0.008
	DBP (mmHg)	78.00 (69.00, 86.00)	78.00 (69.00, 86.00)	78.00 (68.00, 86.00)	0.825
	HR (bpm)	76.00 (68.00, 86.00)	75.00 (66.00, 85.00)	78.00 (70.00, 87.00)	<0.001
Lifestyle factor (n, %)	Smoking history	2024 (53.20)	1296 (53.70)	728 (52.10)	0.346
Past medical history (n, %)	Hypertension	1853 (48.70)	1173 (48.60)	680 (48.70)	0.963
	DM	671 (17.60)	458 (19.00)	213 (15.3)	0.004
	Hyperlipidemia	270 (7.10)	160 (6.60)	110 (7.90)	0.149
	AF	67 (1.80)	43 (1.80)	24 (1.70)	0.886
	HF	10 (0.30)	7 (0.30)	3 (0.20)	0.662
	MI	123 (3.20)	83 (3.40)	40 (2.90)	0.333
	PCI	161 (4.20)	110 (40.60)	51 (3.70)	0.180
	Stroke	222 (5.80)	127 (5.30)	95 (6.80)	0.051

SBP, systolic blood pressure; DBP, diastolic blood pressure; HR, heart rate; DM, diabetes mellitus; AF, atrial fibrillation; HF, heart failure; MI, myocardial infarction; PCI, percutaneous coronary intervention; SII, systemic immune-inflammation index.

### 3.3 Laboratory, Oral Medication, and Angiographic Characteristics

The laboratory, oral medication, and angiographic characteristics of the studied groups are presented in Table 2. No significant differences were found in the TIMI flow grade 3 after pPCI, stent implantation for infarct-related artery (IRA), multivessel disease, dual antiplatelet, and β-blocker therapy. There was a significant difference between the two groups in SII and related indicators (platelet count, neutrophil count, lymphocyte count): (neutrophil count: 6.94 (5.42, 8.57) *vs*. 10.50 (8.72, 12.54); lymphocyte count: 1.52 (1.11, 2.02) *vs*. 0.89 (0.68, 1.15); platelet count: 195.00 (161.00, 232.00) *vs*. 224.00 (189.00, 266.00); SII value: 933.00 (611.48, 1284.63) *vs*. 2486.89 (2042.39, 3263.74)), with their both *p* < 0.001. The percentage of anterior myocardial infarction (45.10% *vs*. 51.60%), IRA-left anterior descending artery (LAD) (45.70% *vs*. 52.00%), TIMI flow grade 0 before pPCI (70.10% *vs*. 75.70%), complete revascularization (58.20% *vs*. 62.80%) were significantly lower in the Q1 group than in the Q2 group (all *p* < 0.05). More importantly, the incidence of new-onset HF (9.60% *vs*. 13.10%) was significantly lower in the Q1 group than in the Q2 group (*p* < 0.001). However, the proportion of angiotensin-converting enzyme inhibitor (ACEI)/angiotensin receptor blocker (ARB)/angiotensin receptor-neprilysin inhibitor (ARNI) (70.10% *vs*. 62.80%) and statin (99.20% *vs*. 98.90%) were higher in the Q1 group (all *p* < 0.05).

**Table 2.** **Laboratory, oral medication, and angiographic characteristics according to SII level strata**.

	Terms	Overall	Q1 (<1707.31)	Q2 (≥1707.31)	*p*-value
		3808	2412	1396	
Laboratory test (×10^9^/L)	Neutrophil count	8.14 (6.24, 10.38)	6.94 (5.42, 8.57)	10.50 (8.72, 12.54)	<0.001
	Lymphocyte count	1.23 (0.88, 1.77)	1.52 (1.11, 2.02)	0.89 (0.68, 1.15)	<0.001
	Platelet count	205.00 (170.00, 245.00)	195.00 (161.00, 232.00)	224.00 (189.00, 266.00)	<0.001
	SII	1337.66 (807.93, 2147.99)	933.00 (611.48, 1284.63)	2486.89 (2042.39, 3263.74)	<0.001
Present status	Symptom onset to balloon time (h)	5.00 (3.00, 7.00)	5.00 (3.00, 8.00)	5.00 (3.00, 7.00)	0.291
	Preoperative malignant arrhythmia (%)	261 (6.90)	162 (6.70)	99 (7.10)	0.659
	Syncope (%)	62 (1.60)	42 (1.70)	20 (1.40)	0.468
	Anterior MI (%)	1808 (47.50)	1087 (45.10)	721 (51.60)	<0.001
Oral medication (%)	Dual antiplatelet therapy	3779 (99.20)	2392 (99.20)	1387 (99.40)	0.528
	Statin	3780 (99.30)	2392 (99.20)	1380 (98.90)	0.024
	β-blocker	3210 (84.30)	2019 (83.70)	1191 (85.30)	0.189
	ACEI/ARB/ARNI	2566 (67.40)	1690 (70.10)	876 (62.80)	<0.001
CAG and PCI (%)	IRA-LAD	1828 (48.00)	1102 (45.70)	726 (52.00)	<0.001
	IRA-LCX	446 (11.70)	326 (13.50)	120 (8.60)	
	IRA-LM	1528 (40.10)	980 (40.60)	548 (39.30)	
	IRA-RCA	6 (0.20)	4 (0.20)	2 (0.10)	
	Multivessel disease	1919 (50.40)	1243 (51.50)	676 (48.40)	0.113
	Stent implantation for IRA	3538 (92.90)	2252 (93.40)	1286 (92.10)	0.149
	Non-IRA PCI management	309 (8.10)	190 (7.90)	119 (8.50)	0.481
	Complete revascularization	2279 (59.80)	1403 (58.20)	876 (62.80)	0.005
	TIMI grade 0 before pPCI	2748 (72.20)	1691 (70.10)	1057 (75.70)	<0.001
	TIMI grade 3 after pPCI	3760 (98.70)	2386 (98.90)	1374 (98.40)	0.184
Outcome (%)	New-onset HF	414 (10.90)	231 (9.60)	183 (13.10)	<0.001

SII, systemic immune-inflammation index; IRA, infarct-related artery; ACEI, angiotensin-converting enzyme inhibitor; ARB, angiotensin receptor blocker; ARNI, angiotensin receptor-neprilysin inhibitor; LAD, left anterior descending artery; LCX, left circumflex artery; RCA, right coronary artery; LM, left main coronary artery; TIMI, thrombolysis in myocardial infarction; PCI, primary percutaneous coronary intervention; MI, myocardial infarction; pPCI, primary percutaneous coronary intervention; HF, heart failure; CAG, coronary angiography.

### 3.4 Correlation between SII and the Risk of New-Onset In-Hospital HF in STEMI Patients after pPCI

Table 3 shows the results of the multivariate regression analysis. We found that there was a correlation between SII and the risk of new-onset in-hospital HF (all *p* trend < 0.05) through unadjusted Model 1: OR = 1.424 (1.159, 1.751); the OR of Q1 was set as 1 to act as the reference. After adjusting for other potential confounding factors, we constructed various models to assess the independent effects of SII on the occurrence of new-onset in-hospital HF in STEMI patients. Model 2 was established after adjusting age, gender, SBP, DBP, and HR, and the Q2 OR was 1.427 (1.155, 1.762). The same result was found in Model 3 after adding several historical factors (smoking, DM, hypertension, hyperlipidemia, AF, and stroke), and the OR was 1.417 (1.145, 1.753) in Q2. Model 4 was further adjusted for the present historical status (symptom onset to balloon time, preoperative malignant arrhythmia, syncope) based on Model 3, and the OR was 1.421 (1.148, 1.759) in Q2. We continued to add pPCI treatment status (TIMI flow grade 0 before pPCI, TIMI flow grade 3 after pPCI, non-infarct related artery lesions PCI management, multivessel disease, infarct-related artery lesions PCI management) for Model 5, and the OR was 1.378 (1.111, 1.709) in Q2. All five Models showed stability in that the risk of new-onset in-hospital HF was higher in Q2 than in Q1 (all *p* < 0.005).

**Table 3.** **Unadjusted and adjusted risk of new-onset in-hospital HF in STEMI patients who underwent pPCI**.

SII level strata	Model 1	Model 2	Model 3	Model 4	Model 5
Q1 adj. OR (95% CI)	1 (reference)	1 (reference)	1 (reference)	1 (reference)	1 (reference)
Q2 adj. OR (95% CI)	1.424 (1.159, 1.751)	1.427 (1.155, 1.762)	1.417 (1.145, 1.753)	1.421 (1.148, 1.759)	1.378 (1.111, 1.709)
*p*-value	0.001	0.001	0.001	0.001	0.004

Model 1 no variables adjusted; Model 2 was adjusted for age, gender, SBP, DBP, and HR; Model 3 was adjusted for age, gender, SBP, DBP, HR, and historical status (smoking, DM, hypertension, hyperlipidemia, AF, and stroke); Model 4 was adjusted for age, gender, SBP, DBP, HR, historical status (smoking, DM, hypertension, hyperlipidemia, AF, and stroke), and present status (symptom onset to balloon time, preoperative malignant arrhythmia, syncope); Model 5 was adjusted for age, gender, SBP, DBP, HR, historical status (smoking, DM, hypertension, hyperlipidemia, AF, and stroke), present status (symptom onset to balloon time, preoperative malignant arrhythmia, syncope), and pPCI treatment status (TIMI flow grade 0 before pPCI, TIMI flow grade 3 after pPCI, non-infarct related artery lesions PCI management, multivessel disease, infarct-related artery lesions PCI management). Patients with an SII level of <1707.31 × 10^9^/L served as a reference group with OR = 1. HF, heart failure; STEMI, ST-segment elevation myocardial infarction; pPCI, primary percutaneous coronary intervention; OR, odds ratio; CI, confidence interval; SBP, systolic blood pressure; DBP, diastolic blood pressure; HR, heart rate; DM, diabetes mellitus; AF, atrial fibrillation; TIMI, thrombolysis in myocardial infarction.

### 3.5 Subgroup Analysis between SII and the Risk of New-Onset In-Hospital HF in Different Ages and Genders

Subgroup analysis (Table 4) of gender was performed by adjusting for certain factors, including age, SBP, DBP, HR, historical status (smoking, DM, hypertension, hyperlipidemia, AF, and stroke), present status (symptom onset to balloon time, preoperative malignant arrhythmia, syncope), and pPCI treatment status (TIMI flow grade 0 before pPCI, TIMI flow grade 3 after pPCI, non-infarct related artery lesions PCI management, multivessel disease, infarct-related artery lesions PCI management). Subsequently, Q2 was found to be 1.505 (1.174, 1.928) times Q1 in males (*p* < 0.001), while Q2 was 1.113 (0.695, 1.783) times Q1 in females, with no statistical difference (*p* = 0.655).

**Table 4.** **The adjusted risk of new-onset in-hospital HF in STEMI patients of different genders and ages who underwent pPCI**.

SII level strata	Gender^*^		Age^#^	
	Male	Female	<60 y	≥60 y
Q1 adj. OR (95% CI)	1 (reference)	1 (reference)	1 (reference)	1 (reference)
Q2 adj. OR (95% CI)	1.505 (1.174, 1.928)	1.113 (0.695, 1.783)	1.135 (0.783, 1.646)	1.525 (1.169,1.989)
*p*-value	0.001	0.655	0.503	0.002

^*^Adjusted for certain factors, including age, SBP, DBP, HR, historical status (smoking, DM, hypertension, hyperlipidemia, AF, and stroke), present status (symptom onset to balloon time, preoperative malignant arrhythmia, syncope), and pPCI treatment status (TIMI flow grade 0 before pPCI, TIMI flow grade 3 after pPCI, non-infarct related artery lesions PCI management, multivessel disease, infarct-related artery lesions PCI management). ^#^Adjusted for certain factors, including gender, SBP, DBP, HR, historical status (smoking, DM, hypertension, hyperlipidemia, AF, and stroke), present status (symptom onset to balloon time, preoperative malignant arrhythmia, syncope), and pPCI treatment status (TIMI flow grade 0 before pPCI, TIMI flow grade 3 after pPCI, non-infarct related artery lesions PCI management, multivessel disease, infarct-related artery lesions PCI management). Patients with an SII level of <1707.31 × 10^9^/L served as a reference group with OR = 1. HF, heart failure; STEMI, ST-segment elevation myocardial infarction; pPCI, primary percutaneous coronary intervention; OR, odds ratio; CI, confidence interval; SBP, systolic blood pressure; DBP, diastolic blood pressure; HR, heart rate; DM, diabetes mellitus; AF, atrial fibrillation; TIMI, thrombolysis in myocardial infarction.

Subgroup analysis of age was performed by adjusting for other factors, including gender, SBP, DBP, HR, historical status (smoking, DM, hypertension, hyperlipidemia, AF, and stroke), present status (symptom onset to balloon time, preoperative malignant arrhythmia, syncope), and pPCI treatment status (TIMI flow grade 0 before pPCI, TIMI flow grade 3 after pPCI, non-infarct related artery lesions PCI management, multivessel disease, infarct-related artery lesions PCI management). We found that Q2 was 1.525 (1.169, 1.989) fold higher of Q1 in older patients (≥60 years; *p* = 0.002).

### 3.6 Sensitivity Analysis after Excluding Patients with Previous HF, MI, or PCI Treatment

We know that patients who have previously experienced HF, MI, or have a history of PCI exhibit a significantly increased risk of postoperative recurrence of HF and mortality after STEMI. Thus, the relationship between SII and the new-onset risk of in-hospital HF was analyzed again after excluding patients who had previously experienced HF, MI, or those who had undergone PCI. Calibration was conducted again for four additional models based on Model 1 (Table 5). The results showed that all models were statistically significant (*p* < 0.05), suggesting that SII was related to the new-onset in-hospital HF risk in STEMI patients who have not experienced HF, MI, or previous PCI.

**Table 5.** **The unadjusted and adjusted risk of new-onset in-hospital HF in STEMI patients who underwent pPCI, excluding previously experienced HF, MI, or previous PCI**.

SII level strata	Model 1	Model 2	Model 3	Model 4	Model 5
Q1 adj. OR (95% CI)	1 (reference)	1 (reference)	1 (reference)	1 (reference)	1 (reference)
Q2 adj. OR (95% CI)	1.373 (1.109, 1.700)	1.383 (1.111, 1.721)	1.375 (1.103, 1.714)	1.375 (1.103, 1.715)	1.330 (1.064, 1.662)
*p*-value	0.004	0.004	0.005	0.005	0.012

Model 1 no variables adjusted; Model 2 was adjusted for age, gender, SBP, DBP, and HR; Model 3 was adjusted for age, gender, SBP, DBP, HR, and historical status (smoking, DM, hypertension, hyperlipidemia, AF, and stroke); Model 4 was adjusted for age, gender, SBP, DBP, HR, historical status (smoking, DM, hypertension, hyperlipidemia, AF, and stroke), and present status (symptom onset to balloon time, preoperative malignant arrhythmia, syncope); Model 5 was adjusted for age, gender, SBP, DBP, HR, historical status (smoking, DM, hypertension, hyperlipidemia, AF, and stroke), present status (symptom onset to balloon time, preoperative malignant arrhythmia, syncope), and pPCI treatment status (TIMI flow grade 0 before pPCI, TIMI flow grade 3 after pPCI, non-infarct related artery lesions PCI management, multivessel disease, infarct-related artery lesions PCI management). Patients with an SII level of <1707.31 × 10^9^/L served as a reference group with OR = 1. HF, heart failure; STEMI, ST-segment elevation myocardial infarction; pPCI, primary percutaneous coronary intervention; OR, odds ratio; CI, confidence interval; SBP, systolic blood pressure; DBP, diastolic blood pressure; HR, heart rate; DM, diabetes mellitus; AF, atrial fibrillation; TIMI, thrombolysis in myocardial infarction; MI, myocardial infarction; PCI, percutaneous coronary intervention.

## 4. Discussion

This study explored the relationship between SII and the risk of new-onset in-hospital HF in 3808 STEMI patients undergoing pPCI. We found a strong correlation between SII and the risk of new-onset in-hospital HF by analyzing patients’ sociodemographic characteristics, vital signs, historical status, present status, and pPCI treatment status. The incidence of new-onset in-hospital HF was elevated in STEMI patients who underwent pPCI with increased inflammatory index SII, especially in male and older patients (≥60 years). To our knowledge, this is the first study to determine the value of SII in STEMI patients who underwent pPCI for the risk of new-onset in-hospital HF.

CAD is a heart disease caused by atherosclerotic (AS) lesions in the coronary arteries, causing vascular narrowing or obstruction, myocardial ischemia, hypoxia, or necrosis [13]. MI, particularly STEMI, is the most pernicious and urgent type of CAD [14]. Atherosclerosis is an inflammatory disease where leucocytes, lymphocytes, and platelets reactivity might have a central role [14, 15]. It was recently shown that inflammatory cells (e.g., white blood cells and subtypes) could be used to estimate prognosis in STEMI patients; however, inflammatory predictors based on 1 to 2 components are relatively poor predictors of prognosis in STEMI [16, 17]. Thus, numerous different studies have recently explored different biomarkers of inflammation, such as neutrophil-to-lymphocyte ratio (NLR), platelet-to-lymphocyte ratio (PLR), and monocyte-to-lymphocyte ratio (MLR), to identify those STEMI patients with higher morbidity and mortality who may benefit from earlier aggressive therapy [6, 18, 19, 20, 21]. The measurable inflammatory markers mentioned above represent local immune responses and inflammation in the progression of STEMI, although inflammation may also be systemic [7].

In 2014, Hu *et al*. [22] reported that the SII is a comprehensive novel inflammatory biomarker that can reflect the local immune response and systemic inflammation of the entire human body. SII is a value calculated from the number of platelets, neutrophils, and lymphocytes in the same blood routine results and through platelet count × neutrophil count/lymphocyte count [22, 23]. The SII index has been widely reported to be associated with poor prognosis in patients with various diseases (fibrosis, cancers, necrotizing enterocolitis, pancreatitis, *etc*.), suggesting that the SII index may serve as a biomarker of future prognosis in multiple diseases [23, 24, 25, 26, 27]. The involvement of inflammatory responses in myocardial ischemia and subsequent reperfusion leads to further damage to cardiomyocytes and, ultimately, HF development and progression [28]. The inflammatory response can lead to lymphocyte apoptosis, causing decreased lymphocyte counts [29, 30], and the inflammatory response can lead to neutrophil and platelet enlargement [31, 32] in the course of acute myocardial infarction and subsequent myocardial ischemia-reperfusion; thus, an elevated SII might be linked to the increased inflammatory activity, thereby leading to poor clinical outcomes in STEMI patients undergoing pPCI.

Candemir *et al*. [33] reported that the SII, an inexpensive and easily measurable laboratory variable, was significantly associated with the severity of CAD in patients with stable angina pectoris. This retrospective single-center study analyzed data from 669 patients with stable CAD who underwent CAG. Yang *et al*. [34] further confirmed that the high and low SII values were positively correlated with major adverse cardiovascular events (MACEs) after PCI and HF during hospitalization in CAD patients by including 5602 CAD patients. Huang *et al*. [7] assessed the utility of the SII in estimating the in-hospital and long-term prognosis of older patients with AMI who received PCI, they found that a lower survival rate in patients with higher SII scores, which also predicted hospital and long-term outcomes. Although their study also compared SII and patient outcomes after pPCI for AMI, they only included 711 older patients from one center. Moreover, the risk of HF after PCI for STEMI has been highlighted in our study.

The present study extends previous knowledge on the association between SII level and new-onset risk in-hospital HF for STEMI patients who underwent pPCI. We concluded that the higher the elevated SII level, the higher the occurrence of the risk of new-onset in-hospital HF, which was consistent with previous related research [7, 33, 34]. The increase in SII in STEMI patients receiving pPCI can be regarded as an inflammation-mediated increase in neutrophils and platelets and an apoptosis-mediated decrease in lymphocytes. The pathogenesis between elevated SII and the risk of new-onset in-hospital HF may be related to calculating SII values for three types of cells: neutrophil, platelet, and lymphocyte. Indeed, neutrophils are the most abundant type of granulocyte, accounting for 40%–70% of all white blood cells in humans. Additionally, neutrophils are the main phagocytes in the blood and play an extremely central role in inflammatory reactions [35]. Neutrophils participate in the whole process of AS, especially in the late stage of AS; they aggravate tissue damage and inflammation by triggering the dissolution and death of smooth muscle cells [36]. Neutrophils have been shown to exacerbate tissue damage and inflammation in advanced stages of atherosclerosis by triggering lysis and death of smooth muscle cells. Sezer *et al*. [37] found that the incidence of microvascular reperfusion injury after coronary arterial blood revascularization in patients with AMI was closely related to an increase in neutrophils in the blood of patients. Following AMI, neutrophils infiltrate the infarcted area, further triggering an inflammatory response and initiating the initial stage of cardiac wound repair, ultimately affecting cardiac remodeling and the occurrence of HF [38]. Lymphocytes are the smallest white blood cells (WBCs) produced by lymphatic organs and are an important cellular component of immune responses. Studies have demonstrated that lower lymphocyte concentrations were associated with atherosclerosis progression and adverse clinical outcomes in patients with AMI and the subsequent reperfusion injury process after revascularization [39, 40, 41]; usually, lymphocytes are relatively reduced after AMI. Platelets are small cytoplasmic pieces separated from the mature megakaryocyte cytoplasm of bone marrow and represent the smallest blood cell. The core function of platelets is prothrombotic potential in arterial thrombosis [42]. In addition, platelets are also involved in inflammation and the formation of AS [43]. Activated platelets secrete many chemokines, which initiate or promote local inflammatory processes at sites of vascular injury and have been shown to play a role in the progression of atherosclerotic lesions and atherothrombosis [42, 43]. As a result, it is believed that platelets may have predictive value for the development of CAD and the clinical prognosis after AMI [44]. In light of the above-mentioned evidence, as a new comprehensive inflammatory marker, the SII involves three inflammatory cell types (neutrophils, lymphocytes, and platelets) and has been argued to be more predictive than simply using 1 to 2 component predictors [33].

Previous studies have shown a positive correlation between long-term prognosis (mortality and major adverse cardiovascular and cerebrovascular events) and SII values in stable CAD patients and patients with acute AMI undergoing pPCI [7, 33]. Our study is the first to find the value of SII in STEMI patients who underwent pPCI for the risk of new-onset in-hospital HF. The five models exhibited consistent OR levels (Model 1: Q2 = 1.424; Model 2: Q2 = 1.427; Model 3: Q2 = 1.417; Model 4: Q2 = 1.421; Model 5: Q2 = 1.378). In subgroup analysis, we found that the relationship between SII and the risk of new-onset in-hospital HF in STEMI patients who underwent pPCI is particularly obvious in males and older individuals. Research reports show differences in inflammatory responses between males and females [45]. The sexual dimorphism observed in inflammatory activity is thought to be driven by the differences in reproductive hormones, with testosterone at play for males, while estrogen modulates inflammatory activity for females [45, 46]. Systemic inflammation is a primary biobehavioral pathway linking sexual and gender stigma to physical health outcomes [47]. Another study has also shown that gender can affect the function of immune cells such as neutrophils and lymphocytes [48]. Our research found a positive association between SII values and the incidence of HF after pPCI in male patients with STEMI; however, this association was not significant in female patients. We believe that the most critical reason may be the relationship between estrogen and androgens.

In addition to grouping by gender, we conducted subgroup analysis based on age. Our study found a positive association between SII values and the incidence of HF after pPCI in older patients (≥60 years) with STEMI, but this association was not significant in relatively young patients (<60 years). Immune and inflammatory responses are associated with age, and the balance between inflammatory and anti-inflammatory responses is more robust in relatively young patients but more prone to be broken in older patients, thus possibly contributing to the age difference in the association between SII and post-pPCI HF in STEMI patients.

## 5. Strengths and Limitations

To our knowledge, this study is the first to investigate the correlation between SII and the risk of new-onset in-hospital HF in STEMI patients who underwent pPCI. Another strength is the exclusion criteria of Killip class ≥2; the inclusion of only Killip 1 patients ensures the accuracy of this study.

However, our study has several limitations. Firstly, we compared only the risk of new-onset in-hospital HF as the outcome. Due to being a multicenter study, we did not collect sufficient data, including the values of HF biomarkers brain natriuretic peptide (BNP) or N-terminal pro-brain natriuretic peptide (NT-proBNP) for all patients and the values of cardiac ejection fraction (EF) using echocardiography. In addition, long-term indicators such as all-cause mortality and MACEs have yet to be collected, and further supplementation is needed in our future work. Secondly, we did not compare SII with other inflammatory indicators such as C-reactive protein (CRP), NLR, PLR, MLR, *etc*. We compared which indicator correlates better with new-onset risk in-hospital HF and long-term efficacy in STEMI patients. This may be more meaningful, but the workload will also be greater. Thirdly, myocardial damage occurred for several hours when STEMI patients arrived at the hospital, meaning we could not record their baseline inflammatory state, which might be important in determining potential confounding factors and accordingly influence the findings of this study. Therefore, our further study will analyze the relationship between SII and MACE in a stable phase. Fourthly, it was conducted only in Hubei Province, which is located in the middle of China. This means that our results may have regional differences and may not apply to everyone, especially for patients from other regions worldwide. Fifthly, we only investigated the correlation between SII and the risk of new-onset in-hospital HF; thus, we will next increase the sample size and follow-up time, observe the correlation between SII and long-term prognosis, and build a risk prediction model. Therefore, prospective studies in larger populations worldwide and multiple indicators are needed to validate our conclusions.

## 6. Conclusions

Elevated SII in STEMI patients who underwent pPCI can be used to identify individuals with an increased risk of new-onset in-hospital HF. This correlation was most apparent in males and older individuals. This result emphasizes that SII should be considered a new-onset in-hospital HF risk marker.

## Availability of Data and Materials

The datasets used and/or analyzed during the current study are available from the corresponding author on reasonable request.
